# Effect of *Lactobacillus plantarum* ZFM4 in *Helicobacter pylori*-infected C57BL/6 mice: prevention is better than cure

**DOI:** 10.3389/fcimb.2023.1320819

**Published:** 2024-01-03

**Authors:** Ying-ying Yu, Ling-yan Wu, Xue Sun, Qing Gu, Qing-qing Zhou

**Affiliations:** ^1^ Department of general practice, The Second Affiliated Hospital, Zhejiang University School of Medicine, Hangzhou, Zhejiang, China; ^2^ The Key Laboratory of Intelligent Preventive Medicine of Zhejiang Province, Hangzhou, Zhejiang, China; ^3^ Key Laboratory for Food Microbial Technology of Zhejiang Province, College of Food Science and Biotechnology, Zhejiang Gongshang University, Hangzhou, Zhejiang, China

**Keywords:** *Lactobacillus plantarum* ZFM4, *Helicobacter pylori*, probiotics, prevention, 16s rRNA sequencing

## Abstract

**Objectives:**

This study was performed to explore the preventive and therapeutic effects of *Lactobacillus plantarum* ZFM4 on H. pylori infections of the stomach tissue in C57BL/6 mice.

**Methods:**

In this study, 40 specific-pathogen-free female C57BL/6 mice were randomly divided into five groups, namely, the control, ZFM4 pretreatment) ZFM4 pretreatment before *H. pylori* infected), model (*H. pylori* infected), triple therapy (*H. pylori* infected and treated with triple therapy), and ZFM4 treatment groups (*H. pylori* infected and treated with ZFM4). The preventive and therapeutic effects of *Lactobacillus plantarum* ZFM4 were evaluated in *H. pylori-*infected C57BL/6 mice by assessing gastric tissue morphology, inflammatory cytokine levels, microbial composition, and microbial diversity.

**Results:**

*Lactobacillus plantarum* ZFM4 was able to survive in low gastric pH and play a role in preventing *H. pylori* infection. This was evident from a reduction in both, the gastric inflammatory response and expression of inflammatory factors caused by *H. pylori* infection. *Lactobacillus plantarum* ZFM4 could also inhibit the growth of *H. pylori* via its beneficial impact on the gastric microbiota.

**Conclusion:**

Our findings suggest that *Lactobacillus plantarum* ZFM4 offers superior preventive effects against *H. pylori* infections when used alone. However, the therapeutic effect on established infections is weaker. Further clinical trials are needed to confirm the specific dosage, duration, and other aspects of administration.

## 1 Introduction

*Helicobacter pylori (H. pylori)* infections are known to be closely related to the development of chronic gastritis, gastric ulcers, gastric mucosa-associated lymphoid tissue lymphomas, and gastric cancer (McColl, 2010). An increase in the prevalence of antibiotic resistance is reducing the eradication rate of *H. pylori* infections (Thung et al., 2016; Savoldi et al., 2018; Megraud et al., 2021). Newer approaches therefore need to be urgently identified for treating these infections (Liang et al., 2022; Luo et al., 2023).

The microecology of the digestive tract is believed to be the basis for many chronic diseases (Pedersen et al., 2016; Qi et al., 2021; de Vos et al., 2022). Certain studies have found that the gastrointestinal microbiota plays an important role in the occurrence and development of gastric cancer (Wang et al., 2014; Stewart et al., 2020). They have also confirmed a gradual change in gastric microbe numbers through the stages of evolution of intestinal type gastric cancer from non-atrophic gastritis and intestinal metaplasia (Aviles-Jimenez et al., 2014). Although this indicates the presence of an interaction between gastric microbes and *H. pylori*, the underlying mechanisms remain unclear. It has been hypothesized that the ingestion of certain active antimicrobial microorganisms could affect *H. pylori* colonization and result in pathological changes in the stomach. The use of probiotics therefore provides a new approach for the prevention and treatment of *H. pylori*-related diseases. Studies have found that certain probiotics can produce anti-*H. pylori* substances, inhibit *H. pylori* colonization, enhance gastric mucosal barrier function, and induce immune regulation (Pan et al., 2016; Zhao et al., 2018; Chen et al., 2019; Song et al., 2019; Liang et al., 2022).

Our research team had previously isolated a lactic acid-producing bacterial strain, namely, *Lactobacillus plantarum* ZFM4, which demonstrated antibacterial activity isolated from pickles. This strain has been deposited at the China Center for Type Culture Collection (strain number: CCTCC M 208077). In our previous studies, *Lactobacillus plantarum* demonstrated characteristics of high acid resistance and thermal stability. Notably, it showed good broad-spectrum bacteriostatic effects in Gram-positive and Gram-negative bacteria. In this context, it is worth noting that most of the previously discovered bacteriocins do not inhibit Gram-negative bacteria. Our findings also showed that it inhibits urease activity and decreases *H. pylori* viability (Jiang et al., 2016; Zhou et al., 2020; Yan et al., 2023).

In this study, we further explored the preventive and therapeutic effects of *Lactobacillus plantarum* ZFM4 on *H. pylori* infections of the stomach tissue in C57BL/6 mice.

## 2 Materials and methods

### 2.1 Bacterial strain and culture condition

The *H. pylori* NCTC11637 strain was frozen at −80**°**C and inoculated on to Colombian blood agar plates (containing sheep blood 7%, trimethoprim 5 mg/L, vancomycin 10 mg/L, polymyxin 2500U/L, and amphotericin 10 mg/L); they were then incubated under micro-aerophilic conditions (85% N2, 10% CO2, and 5% O2) for 48 **h**. The resulting isolate was then washed off with brain heart infusion (BHI) liquid medium to obtain the *H. pylori* NCTC11637 bacterial suspension. This suspension was inoculated into BHI liquid medium (5%) and shaken overnight in a micro-aerophilic environment.

The *Lactobacillus plantarum* ZFM4 strain, that was stored at −80**°**C, was streaked onto De Man, Rogosa, and Sharpe solid agar plates and cultured anaerobically for 24 hours at 37**°**C to ensure revival. After two passages of activation, the strain was transferred to Man, Rogosa, and Sharpe medium and cultured for 18 hours at 37**°**C. The isolate was centrifuged to collect the supernatant liquid for subsequent use.

### 2.2 Animal preparation

Forty specific-pathogen-free female C57BL/6 mice (aged 4–5 weeks) were purchased from the Shanghai SLRC Laboratory Animal Co. LTD. SCXK (Shanghai) 2017-0005 (certificate number: 20170005057469). The license number of the laboratory animal house was SYXK (Zhejiang) 2020-0013. The animal experiments were approved by the Animal Ethics Committee of the Second Affiliated Hospital of the Zhejiang University School of Medicine (number: 2020-79).

The animal room was maintained at a temperature of 24°C and a relative humidity of 50–60%. The mice had free access to food and water and the bedding was changed every two days to ensure cleanliness. Both the drinking water and bedding were sterilized at high pressure and temperature.

As described below, the mice were randomly divided into the five following groups after adaptive feeding for 1 week: control, ZFM4 pretreatment, model, triple therapy, and ZFM4 treatment.

#### 2.2.1 Control group

The mice in this group were administered daily gavage with phosphate buffered saline under normal dietary conditions; after 4 weeks, this was followed by gavage with BHI for 4 weeks.

#### 2.2.2 ZFM4 pretreatment group

This group received gavage with *Lactobacillus plantarum* ZFM4 (400 µl ~10^9^ colony forming units [CFU]/ml) daily; 4 weeks later, this was followed by gavage with *H. pylori* NCTC11637 (500 µL ~10^9^ CFU/ml) every other day for 4 weeks.

#### 2.2.3 Model group

This group was administered gavage with *H. pylori* NCTC11637 (500 µl ~10^9^ CFU/ml) every other day; 4 weeks later, this was followed by gavage with BHI for 4 weeks.

#### 2.2.4 Triple therapy group

This group received gavage with *H. pylori* NCTC11637 (500 µl ~10^9^ CFU/ml) every other day; 4 weeks later, treatment (including omeprazole 0.83 mg/ml, amoxicillin 15.63 mg/ml, and metronidazole 25 mg/ml) was administered by gavage at a dosage of 0.1 ml/kg twice a day for 1 week.

#### 2.2.5 ZFM4 treatment group

This group was administered gavage with *H. pylori* NCTC11637 (500 µl ~10^9^ CFU/ml) every other day; 4 weeks later, this was followed by gavage with *Lactobacillus plantarum* ZFM4 (400 µl ~10^9^ CFU/ml) for 4 weeks.

Prior to administration of gavage, the mice were kept in a fasted state overnight. All cases of *H. pylori* infection were confirmed by fecal antigen detection at 4 weeks after intragastric administration and therapy.

**Figure 1:**
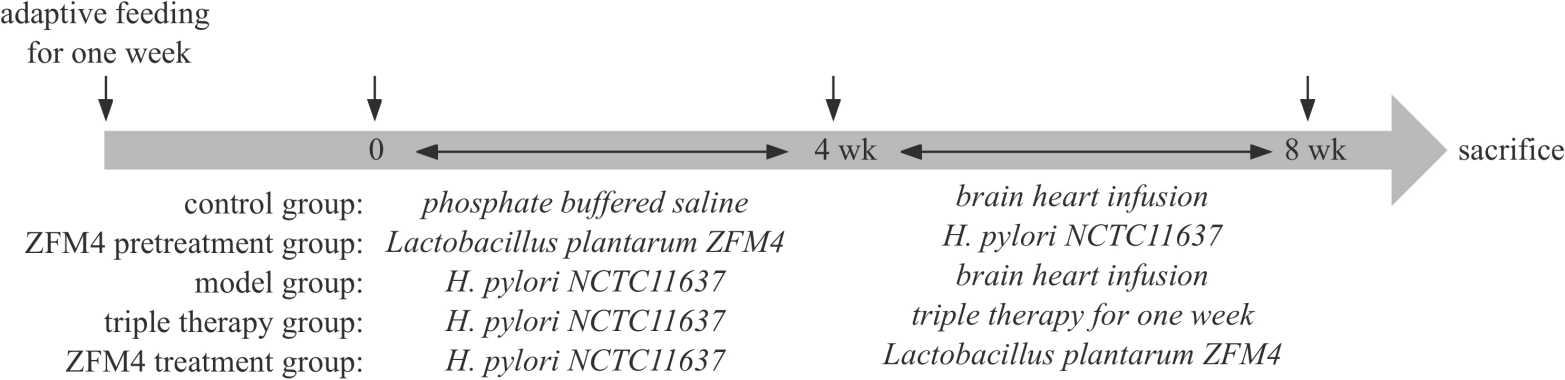
Timeline of the experiment.

No abnormalities were observed during the experiment, which was terminated at week 8. Blood samples were collected from the eyeball and the supernatant was centrifuged and frozen for later use. The mice were sacrificed by decapitation and dissected in a sterile environment, and the antrum was retrieved for future use. The timeline of the experiment was summarized in **Figure 1**.

### 2.3 Colony counts in gastric mucosa

An appropriate quantity of sterile physiological saline was added to gastric mucosal tissue samples obtained from each group. After thorough grinding, the homogenates were diluted at 10 times gradient in sequence. A volume of 1mL of each gastric tissue homogenate was drawn and inoculated on to a new blood agar plate using an L-shaped spreader, which helped to spread the bacterial solution evenly at different angles on each sample plate. The plates were then inverted and placed in a microaerobic incubator for cultivation. Bacterial enumeration and Gram staining were then performed. Results are expressed as mean ± standard deviation.

### 2.4 Histopathological observation of gastric tissue

Inflammation score was according to the visual analogue scale of the updated Sydney system (Dixon et al., 1996) and Chinese consensus on chronic gastritis (2017, Shanghai) (Fang et al., 2018). Three randomly selected fields of view (x 200) were observed under the microscope for each hematoxylin and eosin-stained slice to evaluate infiltration by neutrophils and mononuclear cells. The staining results were evaluated by semi-quantitative analysis: the scores of neutrophil infiltration and mononuclear cells infiltration were combined. Results are expressed as mean ± standard deviation. And the evaluation performed by a trained pathologist who did not know the identity of the tissues observed.

### 2.5 Immunohistochemical analysis

The routine protocol of tissue grossing, processing, paraffin-embedding, and sectioning was followed for the biopsies. The process involved dewaxing, antigen retrieval, and staining with 3,3′-diaminobenzidine and hematoxylin. Immunohistochemical analysis was performed by processing through a gradient of xylene and alcohol, treatment with hydrogen peroxide, and antigen retrieval in citrate buffer. After incubation with primary and secondary antibodies (Abcam PLC, USA), staining and subsequent counterstaining were performed using 3,3′-diaminobenzidine and hematoxylin, respectively. Immunohistochemical results were observed under the microscope. *H. pylori* staining was divided into 4 grades from A to D: grade A: no *H. pylori*; grade B: Occasionally *H. pylori*; grade C: *H. pylori* was scattered or clustered; grade D: a large amout of *H. pylori*.

### 2.6 Interleukin 6 and tumor necrosis factor-α

Enzyme-linked immunosorbent assay kits (Jianglaibio, China) were used to detect the expression levels of interleukin-6 and TNF-α in the samples of venous blood and gastric tissue that were obtained from the C57BL/6 mice in each group.

### 2.7 Microbiota analysis by 16S ribosomal ribonucleic acid gene sequencing

After extracting the total deoxyribonucleic acid from the gastric mucosa samples, primers were designed based on conservative regions and sequencing adapters were added to their ends. Polymerase chain reaction amplification was performed, and the products were purified, quantified, and normalized to form sequencing libraries. The constructed libraries were first subjected to library quality control. Those passing quality control were sequenced using the Illumina Novaseq 6000 system. Information analysis included feature partitioning (operational taxonomic units [OTUs] and amplicon sequence variants), diversity analysis, and differential analysis, among others. Usearch software was used to cluster the reads with a similarity level of 97.0% and obtain OTUs. QIIME software was used for performing beta diversity analysis to compare the similarity in species diversity among different samples. The heatmap of the samples was drawn using R language tools. Differences between two samples could be visually interpreted based on changes in the color gradient.

### 2.8 Statistical analysis

Statistical analysis was performed using the SPSS 20.0 software package. Homogeneity of variance was tested and one-way analysis of variance was performed for multiple group comparisons. Further pairwise comparisons were made using the least significant difference-t test. The staining results were analyzed semi-quantitatively by combining the scores for neutrophil and mononuclear infiltration and the results were presented as the mean ± standard deviation. All *p* values were based on a two-sided test. A *p* value of <0.05 was considered statistically significant.

## 3 Results

### 3.1 Weight of mice

**Figure 2:**
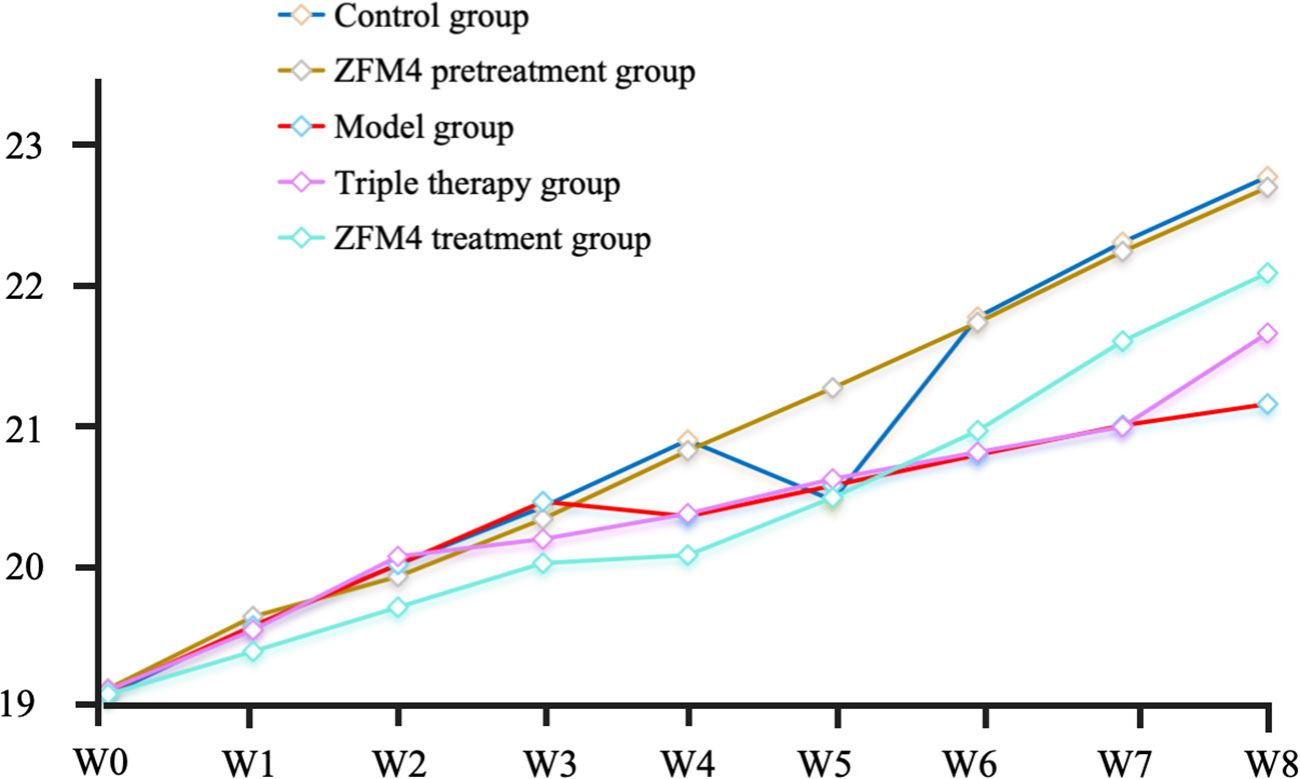
Changes in body weight in each group.

At the end of the experiment, the mice in the control group weighed 22.93 ± 0.76 g. Those in the ZFM4 pretreatment, model, triple therapy, and ZFM4 treatment groups weighed 22.85 ± 0.48, 21.23 ± 0.81, 21.76 ± 0.62, and 22.21 ± 1.02 g, respectively. No statistical difference was observed between the groups (**Figure 2**).

### 3.2 Gastric bacterial colonies among the mice in each group

**Figure 3:**
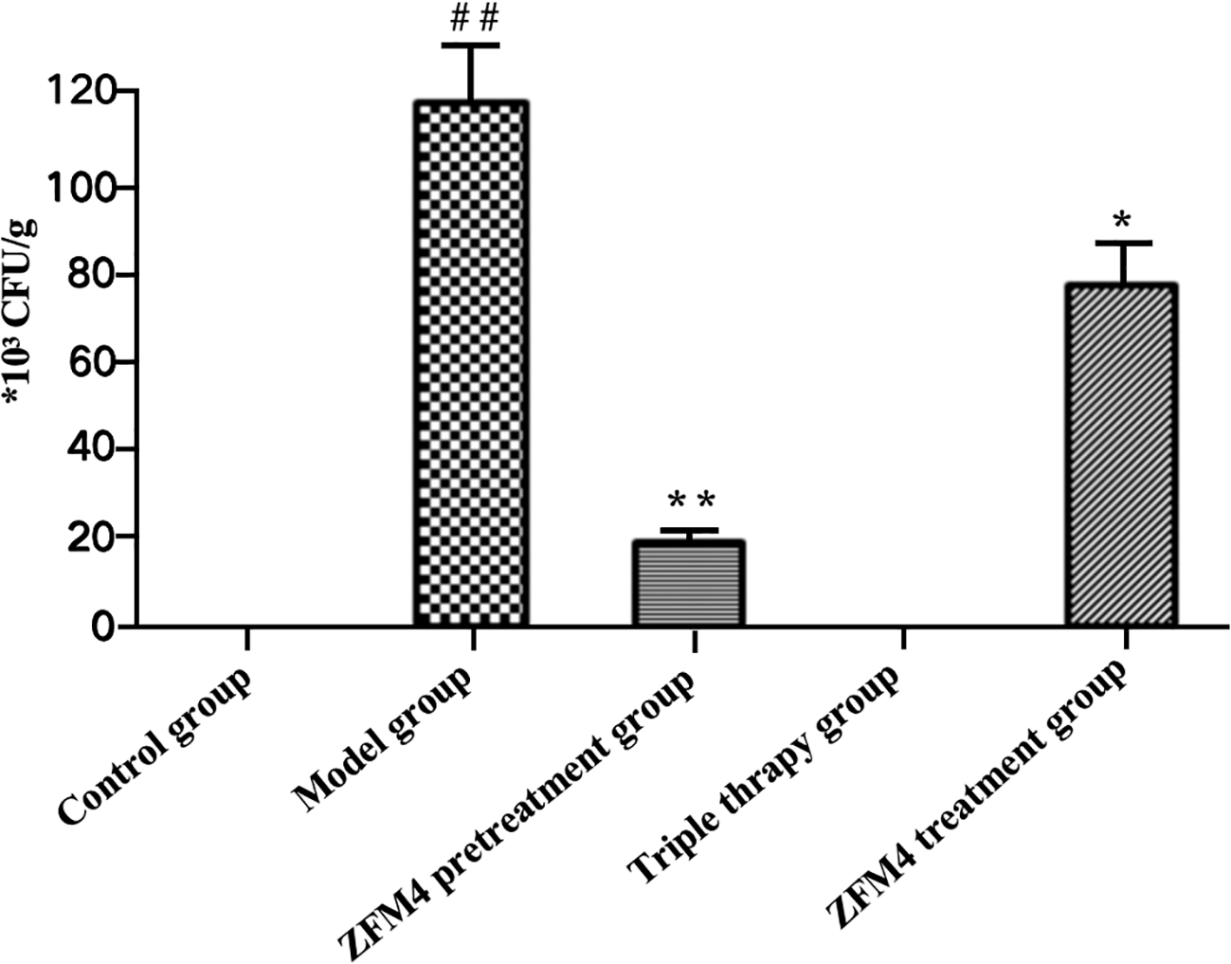
Evaluation of the number of *H. pylori* colonies in each group. Compared with the control group, ##p <0.01. Compared with the model group, **p <0.01, *p <0.05.

**Figure 4:**
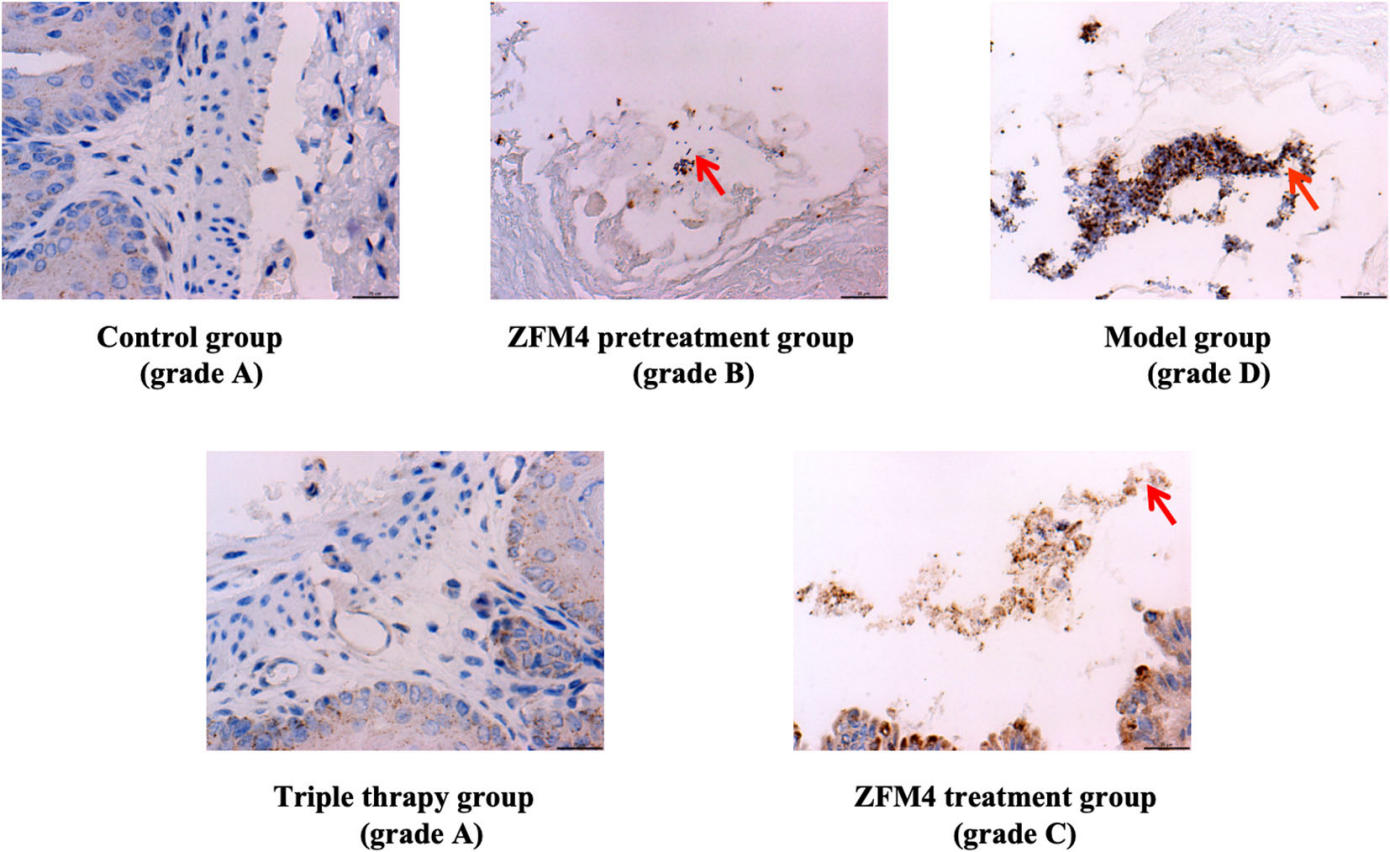
Immunohistochemical analysis of gastric tissue from each group. The red arrows indicate *H. pylori*. *H. pylori* staining was divided into 4 grades from A to D: grade A: no *H. pylori*; grade B: Occasionally *H. pylori*; grade C: *H. pylori* was scattered or clustered; grade D: a large amount of *H. pylori*.

Evaluation of the number of *H. pylori* colonies showed the highest number in the model group with significant statistically difference compared with the control groups (p < 0.01). Both the control and triple therapy groups demonstrated no *H. pylori* colonies. Compared with the model group, the number of *H. pylori* colonies were decreased both in the ZFM4 pretreatment group (p < 0.01) and the ZFM4 treatment group (p < 0.05). However, the colonies of ZFM4 pretreatment group was significantly less than the ZFM4 treatment group (**Figure 3**). Immunohistochemistry provided consistent results (**Figure 4**).

### 3.3 Histopathological evaluation of gastric tissue

**Figure 5:**
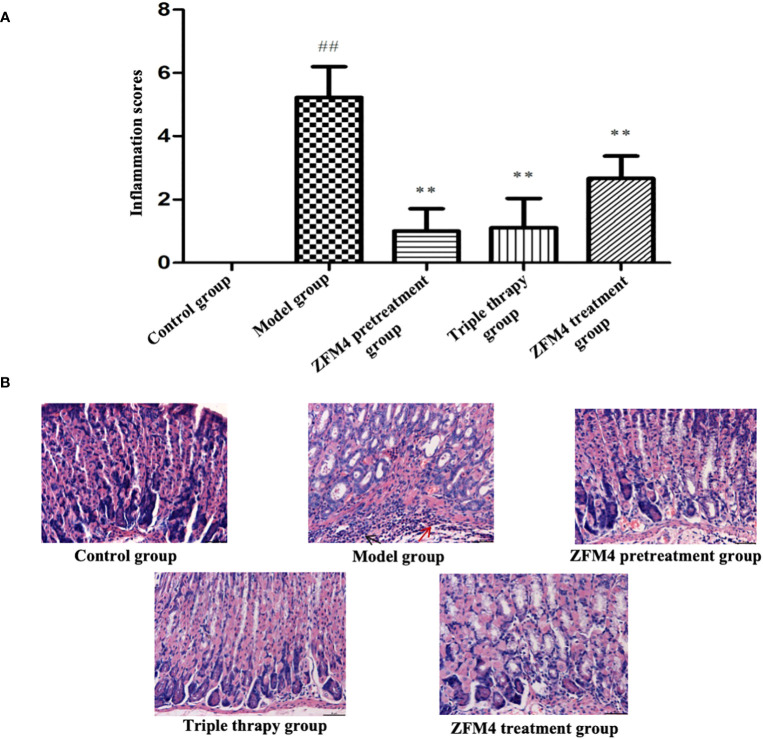
Inflammatory score of gastric tissue sections in each group. Compared with the control group, ##p <0.01. Compared with the model group, **p <0.01, **(A)**. Hematoxylin-Eosin staining of gastric tissue from each group. The red arrow shows neutrophils and the black arrow shows lymphocytes **(B)**.

The model group showed the highest inflammation score after *H. pylori* infection, with a statistically significant difference compared with the control group (p < 0.01). The ZFM4 pretreatment, triple therapy, and ZFM4 treatment groups showed markedly reduced inflammation scores compared with the model group. This difference was statistically significant (p < 0.01). Among the groups that showed a marked reduction in inflammation, the ZFM4 pretreatment group had the lowest inflammation score, followed by the triple therapy group. However, less efficacy was observed in the ZFM4 treatment group (**Figure 5**).

### 3.4 Expression of inflammatory factors in the serum and gastric tissue from each group

**Figure 6:**
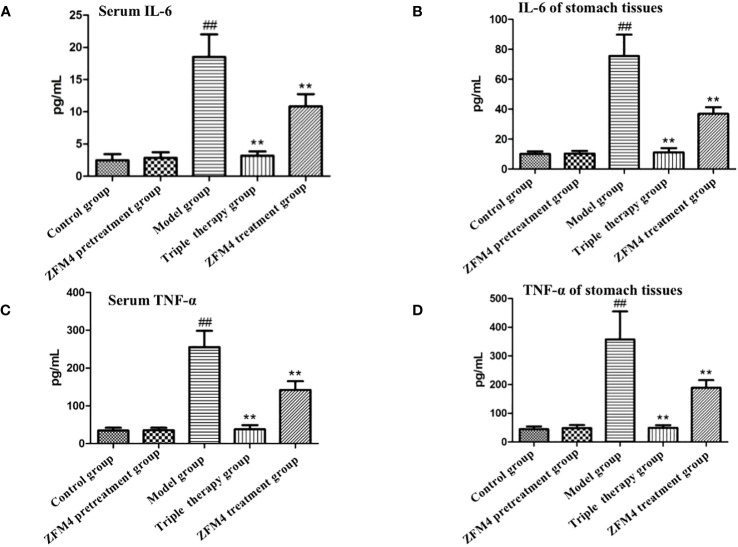
Expression of serum interleukin 6 **(A)**, serum TNF-α **(C)**, gastric interleukin 6 **(B)** and gastric TNF-α **(D)** in each group of mice. ##: compared with the control group, p <0.01; **: Compared with the model, p <0.01.

The serum and gastric tissue samples from each group demonstrated consistent trends in the expression of inflammatory factors. Compared with the control group, the ZFM4 pretreatment group showed no significant change in the expression of inflammatory factors. Conversely, the levels were significantly increased in the model group (P < 0.01). Both the triple therapy and ZFM4 treatment groups showed a significant decrease in expression levels compared with the model group (P < 0.01). However, the ZFM4 treatment group showed greater reduction than the triple therapy group. The levels in the triple therapy group were near the normal level and showed no significant difference compared with the control group (**Figure 6**).

### 3.5 16s sequencing analysis of gastric mucosal microflora structure in each group

**Figure 7:**
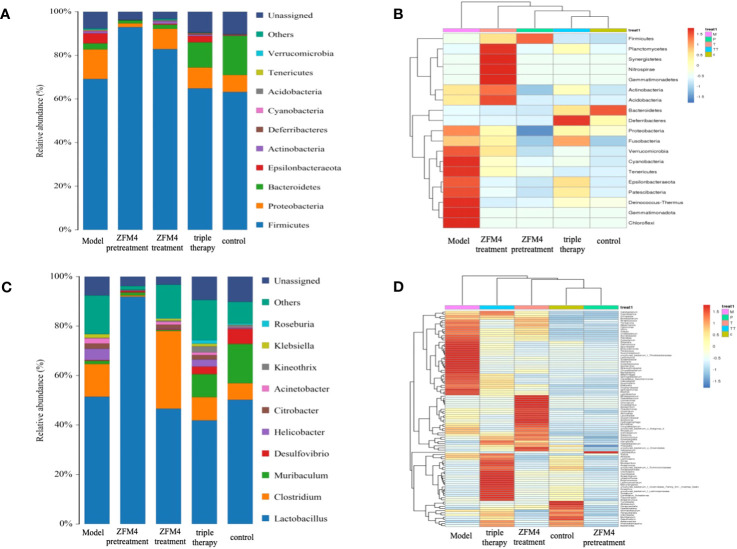
Species distribution map of gastric mucosal bacteria in each group at the phylum level **(A)**. Clustering heatmap of species abundance in each group at the phylum level **(B)**. Species distribution map of gastric mucosal bacteria in each group at the genus level **(C)**. Clustering heatmap of species abundance in each group at the genus level **(D)**. M, Model; P, ZFM4 pretreatment; T, ZFM4 treatment; TT, triple therapy, C, control.

At the phylum level, *Firmicutes*, *Proteobacteria*, and *Bacteroidetes* were the dominant microbiota across all the groups. Among them, *Firmicutes* were the most predominant and accounted for over 70% of the total OTU of each sample (**Figure 7A**). As seen in the clustering heatmap for species abundance (**Figure 7B**), the proportion of *Proteobacteria* was significantly higher in the model group than in the other groups. The ZFM4 pretreatment group had the lowest proportion, indicating the lowest rate of infection with *H. pylori*. However, this group demonstrated the highest proportion of *Firmicutes* among the five groups.

At the genus level, *Lactobacillus plantarum* comprised the highest proportion of the bacterial community (**Figure 7C**). As seen in the clustering heatmap for species abundance (**Figure 7D**), the ZFM4 pretreatment group had the highest proportion of *Lactobacillus plantarum*. Notably, the proportion of *H. pylori* was the lowest in this group and the highest in the model group. Horizontal clustering demonstrated similar species abundance in the different groups. The findings showed that the distance between the ZFM4 pretreatment and control groups was directly proportional to the branch length. This was indicative of similar abundance between the two groups.

**Figure 8:**
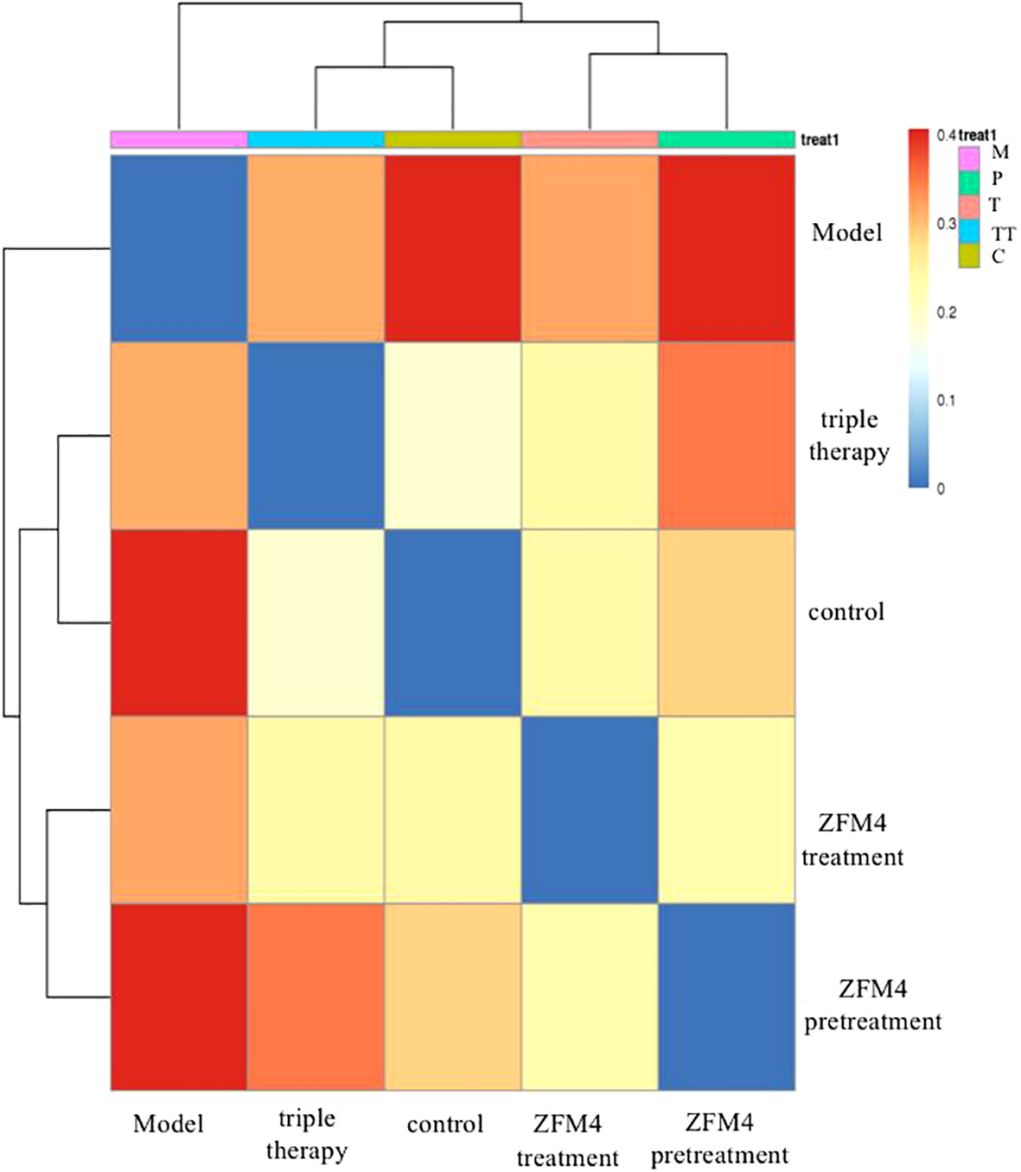
Heat map analysis of gastric mucosal microflora in each group. M, Model; P, ZFM4 pretreatment; T, ZFM4 treatment; TT, triple therapy; C, control.

**Figure 9:**
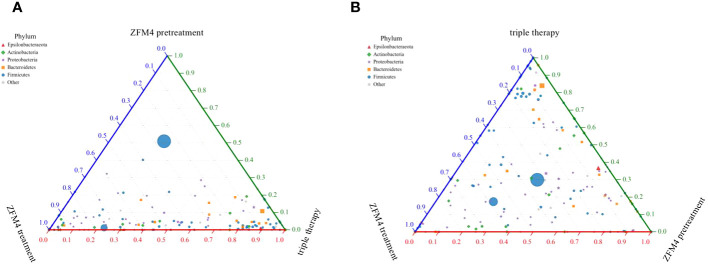
Three phase diagrams for the ZFM4 pretreatment group, ZFM4 treatment group, and triple therapy group at the phylum level.

The differences in species diversity between samples were evaluated using beta diversity analysis. Heatmap analysis showed a considerable difference between the control and model groups in terms of the heat index. The obvious differences between the heat colors were indicative of a significant difference between the overall gastric mucosal microbiota community in these two groups. A significant difference was also observed between the heat index of the ZFM4 pretreatment and model groups. However, the heat index of the ZFM4 pretreatment group was similar to that of the control group (**Figure 8**). Three phase diagrams were used to compare the ZFM4 pretreatment, ZFM4 treatment, and triple therapy groups at the phylum level. The findings indicated that the ZFM4 pretreatment group had the highest proportion of *Firmicutes* (ratio: 5:3:2) and the least *Proteobacteria* (**Figure 9A**). The three phase diagrams used to compare the model, ZFM4 treatment, and triple therapy groups at the phylum level showed the proportion of both *Proteobacteria and Firmicutes* to be higher in ZFM4 treatment group in than the triple therapy group (**Figure 9B**).

## 4 Discussion

For over a century, it was widely believed that the extreme acidic environment of the stomach almost precludes any microbial presence. However, the gastric microbiota gained attention when Marshall and Warren revealed the existence of *H. pylori* in the gastric environment. Modern molecular biology techniques have demonstrated the gastric microbiota to primarily consist of the *Streptococcus*, *Lactobacillus*, and *Propionibacterium* genera (Delgado et al., 2013). Owing to its property of acid resistance, *Lactobacillus* is able to tolerate the acidic environment of the stomach better than other microorganisms and can reach counts of up to 103 CFU/g in a normal stomach. The vast majority of the *Lactobacillus* genus is essential for normal functioning of the human body and is a major source of functional probiotics. Bhatia et al. (1989) were the first to identify a *Lactobacillus* strain that antagonizes *H. pylori*. They also found that short-chain fatty acids are involved in the pathogenesis. Further studies (Sakarya and Gunay, 2014; Qureshi et al., 2019) have suggested that probiotics can compete with *H. pylori* for adhesion receptors and occupy binding sites. They thereby inhibit colonization on the gastric epithelium and reduce the bacterial load. Losurdo G et al (Losurdo et al., 2018). performed a systematic review of 11 studies and found that probiotics may reduce intragastric *H. pylori* charge. Zhao et al. (2018) demonstrated that live cells and supernatants of the probiotic strain Plantarum ZDY2013 could significantly inhibit the activity of *H. pylori* and its urease. They also suggested that this effect may be related to the production of lactic acid, which leads to a decrease in gastric pH. In our study, the results from Gram staining and immunohistochemical evaluation of gastric tissue showed the number of *H. pylori* colonies to be the highest in the model group. This was successively followed by the ZFM4 treatment and ZFM4 pretreatment groups. These results suggest that *Lactobacillus plantarum* ZFM4 had an inhibitory effect on *H. pylori* infections, with better effect in the pretreatment group than in the treatment group.

The second mechanism is that probiotics can interact with gastric mucosal epithelium to regulate the host immune response and promote the secretion of anti-inflammatory cytokines. This improves the inflammatory response and activity of the gastric mucosa (Kim et al., 2008; Turroni et al., 2014; Chen et al., 2019). The results of our study suggest that *Lactobacillus plantarum* ZFM4 can reduce the inflammatory response after *H. pylori* infection, especially when it is administered before the infection. The extent of reduction is better than that of treatment after infection, and is also superior to that of triple therapy. It may therefore be concluded that *Lactobacillus plantarum* ZFM4 offers a certain preventive effect against *H. pylori* infection by reducing the secretion of inflammatory factors.

An increasing number of studies have suggested that probiotics can also inhibit the development of certain “bad” bacterial populations by increasing the diversity or abundance of some “good” bacterial populations. They therefore promote a healthier microbiota in the host. Pan et al. (2016) found that pretreatment with *L. plantarum* ZDY 2013 selectively modulated the gastric microbiota. This may have contributed to a significant preventive effect against *H. pylori* infections. It may have also prevented inflammation of the gastric mucosa and alteration of the gastric microbiota after *H. pylori* infection. Their findings suggested that the oral administration of probiotics that target the gastric microbiota may offer an alternative strategy for preventing *H. pylori* infections. These findings were in agreement with our results.

In our study, 16s sequencing analysis provided a distribution chart of species in the gastric mucosal microflora. At the phylum level, *Firmicutes*, *Proteobacteria*, and *Bacteroidetes* represented the dominant microbiota across all groups. Among them, *Firmicutes* were the most predominant, accounting for over 70% of the total OTU of each sample. *Firmicutes* mainly includes the classes *Bacillus*, *Clostridium*, and *Erysipelotrichia*, among others. The order *Lactobacillales* in class *Bacilli* play a prebiotic role. They promote digestion and maintain the health of the gastrointestinal tract. *Proteobacteria* represent the largest group of bacteria, and include *Escherichia coli*, *Salmonella*, *H. pylori*, and other pathogenic bacteria. This group is an initiator of major gastrointestinal diseases. From the clustering heatmap of species abundance, the proportion of *Proteobacteri*a was found to be significantly higher in the model group than in the other groups. The ZFM4 pretreatment group had the lowest proportion of *Proteobacteria*, indicating the lowest infection rate of *H. pylori.* However, this group had the highest proportion of *Firmicutes* among the five groups. At the genus level, *Lactobacillus plantarum* comprised the highest proportion of the bacterial community. This indicated that it is a major member of the gastrointestinal microbiota and plays an important role in maintaining gut balance. Notably, the ZFM4 pretreatment group had the highest proportion of *Lactobacillus plantarum* and the lowest proportion of *H. pylori*. Conversely, the model group had the highest proportion of *H. pylori.* This suggests that *Lactobacillus plantarum* ZFM4 can effectively inhibit the growth of *H. pylori* via its preventive effects. Beta diversity analysis also indirectly indicated that pretreatment using *Lactobacillus plantarum* ZFM4 (in mice) could ameliorate the changes induced in the gastric microflora by *H. pylori* infections.

The three phase diagrams showed that the ZFM4 pretreatment group had the highest proportion of *Firmicutes* (5:3:2) and the lowest proportion of *Proteobacteria.* The proportions of both these phyla were higher in ZFM4 treatment group than in the triple therapy group. These findings suggest that *Lactobacillus plantarum* ZFM4 offers better preventive effect against *H. pylori* infections when used alone, while its therapeutic effect on established infection is weaker.

In recent years, numerous reports (Poonyam et al., 2019; Zhang et al., 2020; Tang et al., 2021; Moreno Márquez et al., 2022) have recommended the use of probiotics as adjunctive therapy for *H. pylori* infections. In addition to increasing the eradication rate of *H. pylori* infection, probiotics also reduce antibiotic-related adverse reactions. The Maastricht VI/Florence consensus report (Malfertheiner et al., 2022) suggested that further data are needed to assess the direct efficacy of probiotics against *H. pylori* infection. Our findings suggest that the therapeutic effect of *Lactobacillus plantarum* ZFM4 alone is not better than that of standard eradication therapy for *H. pylori*. But *Lactobacillus plantarum* ZFM4 alone exhibited significant advantages for prevention. This is consistent with the findings of an earlier study by Pan et al. (2016).

## 5 Conclusions

In conclusion, *Lactobacillus plantarum* ZFM4 can survive in the low gastric pH and plays a role in preventing *H. pylori* infections. The beneficial effects include a reduction in both the gastric inflammatory response and expression of inflammatory factors related to *H. pylori* infection. It can also inhibit the growth of *H. pylori* via its beneficial impact on the gastric microbiota. Our findings suggest that *Lactobacillus plantarum* ZFM4 offers better preventive effect against *H. pylori* infections when used alone. However, its therapeutic effect is weaker in established infection. Further clinical trials are needed to confirm the specific dosage, duration, and other aspects of administration.

## Data availability statement

The datasets presented in this study can be found in online repositories. The names of the repository/repositories and accession number(s) can be found in the article/supplementary material.

## Ethics statement

The animal study was approved by Animal Ethics Committee of the Second Affiliated Hospital of the Zhejiang University School of Medicine (number: 2020-79). The study was conducted in accordance with the local legislation and institutional requirements.

## Author contributions

YY: Data curation, Formal analysis, Funding acquisition, Investigation, Software, Validation, Visualization, Writing – original draft. LW: Formal analysis, Investigation, Software, Validation, Writing – original draft. XS: Formal analysis, Investigation, Software, Validation, Writing – original draft. QG: Formal analysis, Methodology, Project administration, Writing – review & editing. QZ: Formal analysis, Methodology, Project administration, Writing – review & editing.
